# Cumulant expansion for fast estimate of non-Condon effects in vibronic transition profiles

**DOI:** 10.1038/s41598-017-17506-8

**Published:** 2017-12-14

**Authors:** Joonsuk Huh, Robert Berger

**Affiliations:** 1 Department of Chemistry, Sungkyunkwan University, Suwon, 440-746 Korea; 2 Clemens-Schӧpf Institute, TU Darmstadt, Petersenstr, 22, 64287 Darmstadt, Germany; 3 Frankfurt Institute for Advanced Studies, Goethe University, Ruth-Moufang-Str. 1, 60438 Frankfurt am Main, Germany; 4 Present Address: Fachbereich Chemie, Philipps-Universität Marburg, Hans-Meerwein-Straße 4, 35032 Marburg, Germany

## Abstract

When existing, cumulants can provide valuable information about a given distribution and can in principle be used to either fully reconstruct or approximate the parent distribution function. A previously reported cumulant expansion approach for Franck–Condon profiles [Faraday Discuss., 150, 363 (2011)] is extended to describe also the profiles of vibronic transitions that are weakly allowed or forbidden in the Franck–Condon approximation (non-Condon profiles). In the harmonic approximation the cumulants of the vibronic profile can be evaluated analytically and numerically with a coherent state-based generating function that accounts for the Duschinsky effect. As illustration, the one-photon 1 ^1^A_g_ → 1 ^1^B_2u_ UV absorption profile of benzene in the electric dipole and (linear) Herzberg–Teller approximation is presented herein for zero Kelvin and finite temperatures.

## Introduction

Vibrationally resolved electronic spectra (*e.g*. one-photon absorption and emission spectra) are within the Born-Oppenheimer framework usually interpreted in terms of Franck–Condon (FC) factors (FCFs)^1,2^. Accordingly, one can try to obtain the shape of the spectral profile for a FC-allowed transition from computed FCFs in frequency domain. However, besides the electronic structure calculations, the evaluation of FCFs for large molecular systems is challenging even within the harmonic approximation if one has to take Duschinsky mode mixing (rotation)^3^ into account. This is because multi-variate Hermite polynomials have then to be evaluated for each FC integral, rather than only uni-variate Hermite polynomials as is the case for the comparatively simple parallel harmonic oscillator model. The computational task becomes more difficult as the molecular size and temperature increases because the number of FC integrals grows vastly. The evaluation of the multi-dimensional FC integral in the harmonic approximation is, at least for some cases with Duschinsky mode mixing, classified as a #P-hard problem in computational complexity theory^4^ and thus it recently became a topic in quantum computation. A quantum optical simulation (quantum computation) has been proposed theoretically for instance for the FC profile calculation and has been performed for the photoelectron spectrum of SO_2_ by a trapped-ion device^5–7^.

To describe FC-forbidden or weakly allowed transitions, one has to go beyond the Condon approximation and employ for instance a Herzberg–Teller (HT) expansion^8^ of the electronic transition moment with respect to the normal coordinates. As a result, the calculation of the vibronic spectrum for a non-Condon process is even more difficult than for a FC-allowed transition because one has to evaluate matrix elements of the non-Condon operators which require for each HT integral in general the calculation of combinations of several FC integrals.

The number of FC integrals and matrix elements of non-Condon operators to be evaluated in a sum-over-states approach can, in principle, be significantly reduced with the help of integral configuration selection strategies^9,10^. However, this time-independent (TI) calculation of the spectral profile in the frequency domain is still considerably more expensive than an alternative time-dependent (TD) approach that exploits time-correlation functions (TCFs) (see *e.g*. ref.^11–15^), but offers the ability to directly assign individual peaks in the spectrum. As we have outlined earlier^16^, a unified coherent state-based generating function (CSGF) approach^17^ can be used both for rigorous integral prescreening strategies and TCF calculations which combine the strengths of both approaches and complement each other favorably. Even in the less demanding TD approach, however, one usually invests significant computational time for often unnecessary spectral details.

Cumulants (or moments) of a distribution (see *e.g*. refs^17–27^) can deliver highly useful information. From this one can either attempt to reconstruct the spectral shape or try to estimate the relevant spectral profile, which can be exploited in subsequent TI and TD approaches^16,28^. Cumulants of the vibronic spectrum can be obtained from the CSGF directly without computing the total spectrum in frequency domain. This method was exploited already in ref.^29^ for FC-allowed transitions, and we report herein an extension of this method to incorporate non-Condon transitions. To illustrate the performance of the approach, we present the profile of the ^1^A_1g_ → ^1^B_2u_ transition of benzene, which frequently served as a prototypical example for multiple authors (see *e.g*. refs^30–32^ and references therein). The transition is in the electric dipole approximation Franck–Condon forbidden and it is studied herein at various temperatures within the linear HT and harmonic approximation. Cumulant expansion is compared herein to the TCF approach.

## Results

### Theory

The spectral profile (*ρ*(*ħ* *ω*; *T*)) can be expressed via the Fourier transform (FT) of the TCF (*χ*(*t*; *T*)) that depends on the time *t* and temperature *T*, namely

1 $$\rho (\hslash \omega ;T)={\hslash }^{-1}{\int }_{-\infty }^{\infty }{\rm{d}}{t}\,\chi (t;T){{\rm{e}}}^{i(\omega -{\omega }_{0})t},$$

where *ħω* is the transition energy and *ħω*_0_ the 0′ − 0 transition energy. The corresponding occupancy representation for the TCF and the spectral profile can be obtained from Fermi’s Golden Rule, respectively, as

2 $$\chi (t;T)=\tfrac{\sum _{\underline{v},\underline{v}^{\prime} =\underline{0}}^{\infty }\langle \underline{v}^{\prime} |{\underline{\mu }}^{\dagger }(\underline{Q})|\underline{v}\rangle {\langle \underline{v}^{\prime} |{\underline{\mu }}^{\ast }(\underline{Q})|\underline{v}\rangle }^{\ast }{e}^{-i{E}_{\underline{\varepsilon }^{\prime} ,\underline{\varepsilon }}t/\hslash }{e}^{-\underline{v}\cdot \underline{\varepsilon }/({k}_{{\rm{B}}}T)}}{\prod _{k}{(1-{e}^{-{\varepsilon }_{k}/({k}_{B}T)})}^{-1}}\,,$$

and

3 $$\rho (\hslash \omega ;T)={\textstyle \tfrac{\sum _{\underline{v},\underline{v}^{\prime} =\underline{0}}^{\infty }|\langle \underline{v}^{\prime} |{\mathop{\mu }\limits_{\_}}^{\dagger }(\mathop{Q}\limits_{\_})|\mathop{v}\limits_{\_}\rangle {|}^{2}\delta (\omega -{E}_{\underline{\varepsilon }^{\prime} ,\underline{\varepsilon }}t/\hslash ){e}^{-\mathop{v}\limits_{\_}\cdot \mathop{\varepsilon }\limits_{\_}/({k}_{{\rm{B}}}T)}}{\hslash \prod _{k}{(1-{e}^{-{\varepsilon }_{k}/({k}_{{\rm{B}}}T)})}^{-1}}}\,,$$

where we have assumed an electric dipole transition with the electronic transition dipole moment $(\underline{\hat{\mu }}(\underline{Q}))$, which is a function of normal coordinates of the initial electronic state, and the harmonic approximation. The *N*-dimensional harmonic oscillator eigenstates of the initial and final electronic state are denoted by $|\underline{v}\rangle =|{v}_{1},\ldots ,{v}_{N}\rangle$ and $|\underline{v}^{\prime} \rangle =|{v}_{1}^{\prime} ,\ldots ,{v}_{N}^{\prime} \rangle$ with the corresponding harmonic energy vectors $\underline{\varepsilon }=({\varepsilon }_{1},\ldots ,{\varepsilon }_{N})$ and $\underline{\varepsilon ^{\prime} }=({\varepsilon }_{1}^{\prime} ,\ldots ,{\varepsilon }_{N}^{\prime} )$, respectively. $\hat{H}$ and $\hat{H}\text{'}$ are the *N*-dimensional harmonic oscillator Hamiltonians belonging to the initial and final electronic states, respectively. *k*_B_ is the Boltzmann constant. ${E}_{\underline{\varepsilon }^{\prime} ,\underline{\varepsilon }}$ is the vibronic transition energy with respect to the 0′ − 0 transition energy. The spatial representation of the TCF in closed form can be found by evaluating the following quantum mechanical traces

4 $$\chi (t;T)=\tfrac{{\rm{Tr}}(\underline{\mu }{(\underline{Q})}^{\dagger }\exp (-{\rm{i}}\hat{H}\text{'}t/\hslash )\underline{\mu }(\underline{Q})\exp ({\rm{i}}\hat{H}t/\hslash )\exp (-\hat{H}/({k}_{{\rm{B}}}T)))}{{\mathrm{Tr}(e}^{-\hat{H}/({k}_{{\rm{B}}}T)})}.$$

The traces can be evaluated with any complete basis. In our work *N*-dimensional coherent states were used (see *e.g*. refs^9,16^) with the Duschinsky relation between initial and final state normal coordinates ($\underline{Q}^{\prime} ={\bf{S}}\underline{Q}+\underline{d}$ where ***S*** and $\underline{d}$ are the Duschinsky rotation matrix and displacement vector, respectively and $\underline{Q}^{\prime}$ are the normal coordinates of the final state) and the linear HT expansion of the electronic transition dipole moment ($\underline{\mu }\simeq \underline{\mu }(\underline{0})+{\sum }_{k}{\underline{\mu }}_{k}^{\prime} {\hat{Q}}_{k}$ where ${\underline{\mu }}_{k}^{\prime}$ is the first derivative of $\underline{\mu }$ with respect to ${\hat{Q}}_{k}$.).

The TCF is related to a probability density function (PDF). If all cumulants or moments of a PDF are defined and available, the PDF can be reconstructed as follows^29^

5 $$\chi (t,T)={\rho }_{{\rm{t}}{\rm{o}}{\rm{t}}}\exp (\sum _{k=1}^{{\rm{\infty }}}\frac{{\langle {E}_{\underline{\varepsilon }^{\prime} ,\mathop{\varepsilon }\limits_{\_}}^{k}\rangle }^{{\rm{c}}}(T)}{k!}{({\rm{i}}t/\hslash )}^{k}),$$

where ${\langle {E}_{\underline{\varepsilon }^{\prime} ,\underline{\varepsilon }}^{k}\rangle }^{{\rm{c}}}(T)$ is the *k*-th order cumulant at temperature *T*. The cumulants of the spectral density function are normalised to the total integrated profile ${\rho }_{{\rm{tot}}}=|\underline{\mu }(\underline{0}){|}^{2}+{\sum }_{k}^{N}\frac{{\hslash }^{2}}{2{\varepsilon }_{k}}|{\underline{\mu }}_{k}^{\prime} {|}^{2}\,\coth (\frac{{\varepsilon }_{k}}{2{k}_{B}T})$ ^16^. Moments (cumulants and moments are inter-convertible^33^) can be obtained by partial derivatives of *χ* with respect to time,

6 $$\langle {E}_{\underline{\varepsilon }^{\prime} ,\mathop{\varepsilon }\limits_{\_}}^{k}\rangle (T)={(-\frac{\hslash }{{\rm{i}}})}^{k}\frac{{{\rm{\partial }}}^{k}}{{\rm{\partial }}{t}^{k}}\chi (t;T){|}_{t=0}.$$

The cumulants can be obtained from the moments via the following transformation^33^,

7 $${\langle {E}_{\underline{\varepsilon }^{\prime} ,\mathop{\varepsilon }\limits_{\_}}^{n+1}\rangle }^{{\rm{c}}}=\langle {E}_{\underline{\varepsilon }^{\prime} ,\mathop{\varepsilon }\limits_{\_}}^{n+1}\rangle -\sum _{k=0}^{n-1}(\begin{array}{c}n\\ k\end{array})\langle {E}_{\underline{\varepsilon }^{\prime} ,\mathop{\varepsilon }\limits_{\_}}^{n-k}\rangle {\langle {E}_{\underline{\varepsilon }^{\prime} ,\mathop{\varepsilon }\limits_{\_}}^{k+1}\rangle }^{{\rm{c}}}.$$

Thus, cumulants can be evaluated analytically or numerically by evaluating partial derivatives of *χ* in Eq. (4) with respect to the time variable at *t* = 0. Analytic evaluation of the cumulants to arbitrary order within the linear HT approximation can be performed along the lines of the development in refs^16,28,29^ for the cumulants of FC profiles to arbitrary order. For numerical evaluation of low-order cumulants one needs to compute *χ* at the first few time steps. To obtain the corresponding moments numerically, Re(*χ*(*t*, *T*)) and Im(*χ*(*t*, *T*)) as computed at these time steps are used to determine low-order even and odd moments, respectively, because ${{\rm{e}}}^{-{\rm{i}}{E}_{\underline{\varepsilon }^{\prime} ,\mathop{\varepsilon }\limits_{\_}}t/\hslash }=\,\cos ({E}_{\underline{\varepsilon }^{\prime} ,\mathop{\varepsilon }\limits_{\_}}t/\hslash )-{\rm{i}}\,{\rm{s}}{\rm{i}}{\rm{n}}({E}_{\underline{\varepsilon }^{\prime} ,\mathop{\varepsilon }\limits_{\_}}t/\hslash )$ in Eq. (2) (see *e.g*. ref.^23^).

The closed form of *χ*(*t*, *T*) within the linear HT approximation can be found in refs^14–16,28^. In the present work, flexibility is used in the GF to obtain detailed information concerning individual contributions of different modes. This is achieved by assigning different time and temperature variables to each vibrational degree of freedom. The corresponding GF in an occupancy representation reads as follows

8 $$\begin{array}{rcl}{G}^{K}{({\bf{Z}};\tilde{{\boldsymbol{\Gamma }}})}^{(\hat{f},\hat{g})} & = & {\mathscr{N}}|\langle \underline{0}^{\prime} |\underline{0}\rangle {|}^{-2}\sum _{\underline{v},\underline{v}\text{'}=\underline{0}}^{\underline{\infty }}\langle \underline{v}\text{'}|\hat{f}|\underline{v}\rangle {\langle \underline{v}^{\prime} |\hat{g}|\underline{v}\rangle }^{\ast }\\  &  & \prod _{k\mathrm{=1}}^{N}[{z}_{k}^{2{v}_{k}}{({z}_{k}^{\prime} )}^{2{v}_{k}^{\prime} }]{{\rm{e}}}^{-({\underline{v}}^{{\rm{t}}}{\bf{B}}\underline{\varepsilon }+\underline{v}{\text{'}}^{{\rm{t}}}{\bf{B}}^{\prime} \underline{\varepsilon }^{\prime} )}\,,\end{array}$$

where the general operators $\hat{f}$ and $\hat{g}$, which can be products of momentum and position operators, are given instead of $\underline{\hat{\mu }}(\underline{\hat{Q}})$; and different temperatures can be given to the initial and final vibrational degrees of freedom via

9 $${\bf{B}}={\rm{diag}}({\beta }_{1},\ldots ,{\beta }_{N}\mathrm{),\ }{\bf{B}}^{\prime} ={\rm{diag}}({\beta }_{1}^{\prime} ,\ldots ,{\beta }_{N}^{\prime} ),$$

where *β*_*k*_ = 1/(*k*_B_ *T*_*k*_) (with Boltzmann constant *k*_B_ and temperature *T*_*k*_). The parameter matrices are defined as follows

10 $${\bf{Z}}=(\begin{array}{cc}{\bf{z}} & {\bf{0}}\\ {\bf{0}} & {\bf{z}}^{\prime} \end{array})\,,$$

with the time variables being assigned to the matrices ${\rm{z}}={\rm{diag}}({{\rm{e}}}^{{\rm{i}}{\varepsilon }_{1}{t}_{1}\mathrm{/(2}\hslash )},\ldots ,{{\rm{e}}}^{{\rm{i}}{\varepsilon }_{N}{t}_{N}\mathrm{/(2}\hslash )})$ and ${\rm{z}}^{\prime} ={\rm{diag}}({{\rm{e}}}^{-{\rm{i}}{\varepsilon }_{1}^{\prime} {t}_{1}^{\prime} \mathrm{/(2}\hslash )},$ $\ldots ,{{\rm{e}}}^{-{\rm{i}}{\varepsilon }_{N}^{\prime} {t}_{N}^{\prime} \mathrm{/(2}\hslash )})$ for initial and final vibrational modes, respectively, and

11 $$\tilde{{\boldsymbol{\Gamma }}}=(\begin{array}{cc}{\boldsymbol{\Gamma }} & {\bf{0}}\\ {\bf{0}} & {\boldsymbol{\Gamma }}^{\prime} \end{array})\,,$$

with $${\boldsymbol{\Gamma }}={\rm{diag}}({{\rm{e}}}^{-{\beta }_{1}{\varepsilon }_{1}\mathrm{/2}},\ldots ,{{\rm{e}}}^{-{\beta }_{N}{\varepsilon }_{N}\mathrm{/2}}),{\boldsymbol{\Gamma }}^{\prime} ={\rm{diag}}({{\rm{e}}}^{-{\beta }_{1}^{\prime} {\varepsilon }_{1}^{\prime} \mathrm{/2}},\ldots ,{{\rm{e}}}^{-{\beta }_{N}^{\prime} {\varepsilon }_{N}^{\prime} \mathrm{/2}})$$.

${\mathscr{N}}$ is the corresponding normalizing factor related to the partition function of the Boltzmann distribution of harmonic oscillators, i.e.

12 $${\mathscr{N}}=\prod _{k}^{N}\mathrm{(1}-{{\rm{e}}}^{-{\beta }_{k}{\varepsilon }_{k}}\mathrm{)(1}-{{\rm{e}}}^{-{\beta }_{k}^{\prime} {\varepsilon }_{k}^{\prime} }\mathrm{).}$$

The Duschinsky relation is considered with the Doktorov matrices and vectors^34^

13 $${\bf{W}}=(\begin{array}{cc}{\bf{I}}-2{\bf{Q}} & -2{\bf{R}}\\ -2{{\bf{R}}}^{{\rm{t}}} & {\bf{I}}-2{\bf{P}}\end{array})\,,\quad \underline{r}=\sqrt{2}(\begin{array}{c}-{\bf{R}}\underline{\delta }\\ ({\bf{I}}-{\bf{P}})\underline{\delta }\end{array})\,,$$

14 $$\begin{array}{c}{\bf{Q}}={({\bf{I}}+{{\bf{J}}}^{{\rm{t}}}{\bf{J}})}^{-1}\,,\quad {\bf{P}}={\bf{J}}{\bf{Q}}{{\bf{J}}}^{{\rm{t}}}\,,\\ {\bf{R}}={\bf{Q}}{{\bf{J}}}^{{\rm{t}}}\,,\quad {\bf{J}}={\boldsymbol{\Omega }}^{\prime} {\bf{S}}\,{{\boldsymbol{\Omega }}}^{-1}\,,\quad \underline{\delta }={\boldsymbol{\Omega }}^{\prime} \underline{d}/\sqrt{\hslash }\,,\end{array}$$

as well as

15 $$\begin{array}{c}{\boldsymbol{\Omega }}={\rm{diag}}{(\underline{\varepsilon })}^{\mathrm{1/2}}/\sqrt{\hslash },\\ {\boldsymbol{\Omega }}^{\prime} ={\rm{diag}}{(\underline{\varepsilon }^{\prime} )}^{\mathrm{1/2}}/\sqrt{\hslash }\,\mathrm{.}\end{array}$$

The temperature dependent parameters are defined as follow

16 $${{\bf{W}}}_{T}=\tilde{{\boldsymbol{\Gamma }}}{\bf{W}}\tilde{{\boldsymbol{\Gamma }}},\quad {\underline{r}}_{T}=\tilde{{\boldsymbol{\Gamma }}}\underline{r}\mathrm{.}$$

Finally, the Franck–Condon Herzberg–Teller (FCHT) TCF reads as

17 $$\begin{array}{rcl}\frac{{\chi }_{{\rm{FCHT}}}({\bf{Z}};\tilde{{\boldsymbol{\Gamma }}})}{|\langle \underline{0}^{\prime} |\underline{0}\rangle {|}^{2}} & = & |{\underline{\mu }}_{0}{|}^{2}{G}^{K}({\bf{Z}};\tilde{{\boldsymbol{\Gamma }}})\\  &  & +2\sum _{i}{\underline{\mu }}_{0}\cdot {\underline{\mu }}_{i}^{\prime} {G}^{K}{({\bf{Z}};\tilde{{\boldsymbol{\Gamma }}})}^{({\hat{Q}}_{i},\hat{1})}\\  &  & +\sum _{i,j}{\underline{\mu }}_{i}^{\prime} \cdot {\underline{\mu }}_{j}^{\prime} {G}^{K}{({\bf{Z}};\tilde{{\boldsymbol{\Gamma }}})}^{({\hat{Q}}_{i},{\hat{Q}}_{j})}\,,\end{array}$$

with the FC generating function,

18 $$\begin{array}{c}{G}^{K}({\bf{Z}};\mathop{{\boldsymbol{\Gamma }}}\limits^{ \sim })={G}^{K}{({\bf{Z}};\mathop{{\boldsymbol{\Gamma }}}\limits^{ \sim })}^{(\hat{1},\hat{1})}\\ {\mathscr{N}}det{({\bf{I}}+{\bf{Z}}{{\bf{W}}}_{T}{\bf{Z}})}^{-1/2}det{({\bf{I}}-{\bf{Z}}{{\bf{W}}}_{T}{\bf{Z}})}^{-1/2}\\ \exp (({\mathop{r}\limits_{\_}}_{T}{)}^{{\rm{t}}}{\bf{Z}}{({\bf{I}}+{\bf{Z}}{{\bf{W}}}_{T}{\bf{Z}})}^{-1}{\bf{Z}}{\mathop{r}\limits_{\_}}_{T}),\end{array}$$

the mixed FC/HT generating function,

19 $$\begin{array}{rcl}{G}^{K}{({\bf{Z}};\tilde{{\boldsymbol{\Gamma }}})}^{({\hat{Q}}_{i},\hat{1})} & = & \sqrt{\tfrac{\hslash }{\mathrm{2(}{\varepsilon }_{i}/h)}}{G}^{K}({\bf{Z}};\tilde{{\boldsymbol{\Gamma }}})\\  &  & {[\underline{r}+({\bf{I}}-{\bf{W}})\tilde{{\boldsymbol{\Gamma }}}{\bf{Z}}{({\bf{I}}+{\bf{Z}}{{\bf{W}}}_{T}{\bf{Z}})}^{-1}{\bf{Z}}{\underline{r}}_{T}]}_{i}\,,\end{array}$$

and the HT generating function,

20 $$\begin{array}{rcl}{G}^{K}{({\bf{Z}};\tilde{{\boldsymbol{\Gamma }}})}^{({\hat{Q}}_{i},{\hat{Q}}_{j})} & = & \tfrac{\hslash }{2}\sqrt{\tfrac{1}{({\varepsilon }_{i}/h)({\varepsilon }_{j}/h)}}{G}^{K}({\bf{Z}};\tilde{{\boldsymbol{\Gamma }}})\\  &  & [[\underline{r}+({\bf{I}}-{\bf{W}})\tilde{{\boldsymbol{\Gamma }}}{\bf{Z}}{({\bf{I}}+{\bf{Z}}{{\bf{W}}}_{T}{\bf{Z}})}^{-1}{\bf{Z}}{\underline{r}}_{T}{]}_{i}\\  &  & \times {[\underline{r}+({\bf{I}}-{\bf{W}})\tilde{{\boldsymbol{\Gamma }}}{\bf{Z}}{({\bf{I}}+{\bf{Z}}{{\bf{W}}}_{T}{\bf{Z}})}^{-1}{\bf{Z}}{\underline{r}}_{T}]}_{j}\\  &  & +\tfrac{1}{2}{[({\bf{I}}-{\bf{W}})\tilde{{\boldsymbol{\Gamma }}}{\bf{Z}}{({\bf{I}}+{\bf{Z}}{{\bf{W}}}_{T}{\bf{Z}})}^{-1}{\bf{Z}}\tilde{{\boldsymbol{\Gamma }}}({\bf{I}}-{\bf{W}})]}_{ij}\\  &  & +\tfrac{1}{2}{[({\bf{I}}-{\bf{W}})\tilde{{\boldsymbol{\Gamma }}}{\bf{Z}}{({\bf{I}}-{\bf{Z}}{{\bf{W}}}_{T}{\bf{Z}})}^{-1}{\bf{Z}}\tilde{{\boldsymbol{\Gamma }}}({\bf{I}}-{\bf{W}})]}_{ji}]\,\mathrm{.}\end{array}$$

The electronic 1 ^1^A_g_ → 1 ^1^B_2u_ transition of benzene is FC-forbidden in the electric dipole approximation $(\underline{\mu }(\underline{0})=\underline{0})$ such that only the HT terms contribute to the spectral function. The corresponding TCF for FCHT weighted density of states (FCHTW) is given as follows, here with same time (*t*) for all vibrational degrees of freedom and same temperature (*T*_*k*_ = *T* and *T*_*k*_′ = ∞) for the initial and final modes, respectively,

21 $${\chi }_{{\rm{FCHTW}}}(t;T)=|\langle \underline{0}^{\prime} |\underline{0}\rangle {|}^{2}\sum _{i,j}{\underline{\mu }}_{i}^{\prime} \cdot {\underline{\mu }}_{j}^{\prime} G{(t;T)}^{({\hat{Q}}_{i},{\hat{Q}}_{j})}\mathrm{.}$$

Accordingly, the spectrum is obtained by the Fourier transformation,

22 $${\rho }_{{\rm{FCHTW}}}(\hslash \omega ;T)={\hslash }^{-1}{\int }_{-\infty }^{\infty }{\rm{d}}t\,{\chi }_{{\rm{FCHTW}}}(t;T){{\rm{e}}}^{{\rm{i}}(\omega -{\omega }_{0})t},$$

which can show the detailed vibronic structure.

The FCHTW profile is now approximated with a finite number of cumulants via the Edgeworth expansion with the order *n* ^35^. Whereas for *n* = 2 a Gaussian distribution function is used, the Edgeworth expansion for order *n* ≥ 3 is employed as^29^

23 $$\begin{array}{ccc}{\rho }_{{\rm{F}}{\rm{C}}{\rm{H}}{\rm{T}}{\rm{W}}}^{({\rm{c}})}(\mathop{\nu }\limits^{ \sim };T;n\ge 3) & = & \frac{{\rho }_{{\rm{t}}{\rm{o}}{\rm{t}}}}{\sqrt{2\pi {(h{c}_{0})}^{-2}{\langle {E}_{\underline{\varepsilon }^{\prime} ,\mathop{\varepsilon }\limits_{\_}}^{2}\rangle }^{{\rm{c}}}(T)}}\exp (-\frac{{(\mathop{\nu }\limits^{ \sim }-{\mathop{\nu }\limits^{ \sim }}_{0})}^{2}}{2(h{c}_{0}{)}^{-2}{\langle {E}_{\underline{\varepsilon }^{\prime} ,\mathop{\varepsilon }\limits_{\_}}^{2}\rangle }^{{\rm{c}}}(T)})\\  &  & \times \,[1+\sum _{s=1}^{n}{(\sqrt{{(h{c}_{0})}^{-2}{\langle {E}_{\underline{\varepsilon }^{\prime} ,\mathop{\varepsilon }\limits_{\_}}^{2}\rangle }^{{\rm{c}}}(T)})}^{s}\\  &  & \times \,\sum _{\{\mathop{k}\limits_{\_}\}}{{\mathscr{H}}}_{s+2r}(\mathop{\nu }\limits^{ \sim }-{\mathop{\nu }\limits^{ \sim }}_{0})\prod _{m=1}^{s}\frac{1}{{k}_{m}!}{(\frac{{{\mathscr{S}}}_{m+2}(T)}{(m+2)!})}^{{k}_{m}},]\end{array}$$

where $\{\underline{k}\}$ is a set of non-negative integer vectors, which are constrained to $s={\sum }_{m\mathrm{=1}}^{s}m{k}_{m}$ and $r={\sum }_{m\mathrm{=1}}^{s}{k}_{m}$. ${ {\mathcal H} }_{s+2r}$ is a uni-variate Hermite polynomial of order *s* + 2*r* and ${{\mathscr{S}}}_{m+2}$ is defined as follows,

24 $${{\mathscr{S}}}_{m+2}(T)=\frac{{(h{c}_{0})}^{-(m+2)}{\langle {E}_{\underline{\varepsilon }^{\prime} ,\mathop{\varepsilon }\limits_{\_}}^{m+2}\rangle }^{{\rm{c}}}(T)}{{((h{c}_{0}{)}^{-2}{\langle {E}_{\underline{\varepsilon }^{\prime} ,\mathop{\varepsilon }\limits_{\_}}^{2}\rangle }^{{\rm{c}}}(T))}^{m+1}}.$$

The Edgeworth expansion with a finite number of cumulants and in the infinite series are related as

25 $$\mathop{\mathrm{lim}}\limits_{n\to \infty }{\rho }_{{\rm{FCHTW}}}^{({\rm{c}})}(\tilde{\nu };T;n)={\rho }_{{\rm{FCHTW}}}(\tilde{\nu };T),$$

and we use the relation, ${\rho }_{{\rm{FCHTW}}}(\tilde{\nu };T)=h{c}_{0}{\rho }_{{\rm{FCHTW}}}(\hslash \omega ;T)$, for the wavenumber $(\tilde{\nu })$ domain profile.

### Computational detail

The vibronic profiles for benzene’s 1 ^1^A_g_ → 1 ^1^B_2u_ transition at zero Kelvin and finite temperatures are calculated with the two methods, namely TCF and time-independent cumulant expansion (CE). We use herein the term time-independent CE, which was employed in ref.^29^, to distinguish this CE from the conventional (time-dependent) CE (see *e.g*. refs^20–24^) which involves time integration for the cumulant calculation. To compute the vibronic spectra via the TCF method, the FFTW library^36^ is used for fast Fourier Transform (FFT). The approximate curves are generated for the CE with Edgeworth expansion^29,35^ using the computed low-order cumulants. Some of the problems related to this type of expansion for the description of FC profiles are discussed in ref.^29^. The moments (Eq. 6) are calculated both analytically and numerically for comparison, the latter by approximating partial derivatives of *χ* in Eq. 4 with respect to time (see results section) via a central finite difference scheme with a truncation error being of order (Δ*t*)^2^. When generating the data points in time, we exploit the time-reversal symmetry condition, i.e. *χ*(−*t*; *T*) = *χ*(*t*; *T*)^*^. Required input data from electronic structure calculations for benzene, *i.e*. molecular equilibrium structures and corresponding harmonic force fields for each electronic state (^1^A_g_ and ^1^B_2u_) as well as first derivatives of the electronic transition dipole moments are taken from ref.^30^ (CASSCF/DZV). These data have been compared to results obtained via analytical derivative techniques for electronic transition dipole moments within a time-dependent density functional theory framework in ref.^32^. The vibronic structure methods employed in the present work are implemented in a development version of our vibronic structure program package hotFCHT^9,30^.

### Numerical simulation

**Figure 1:**
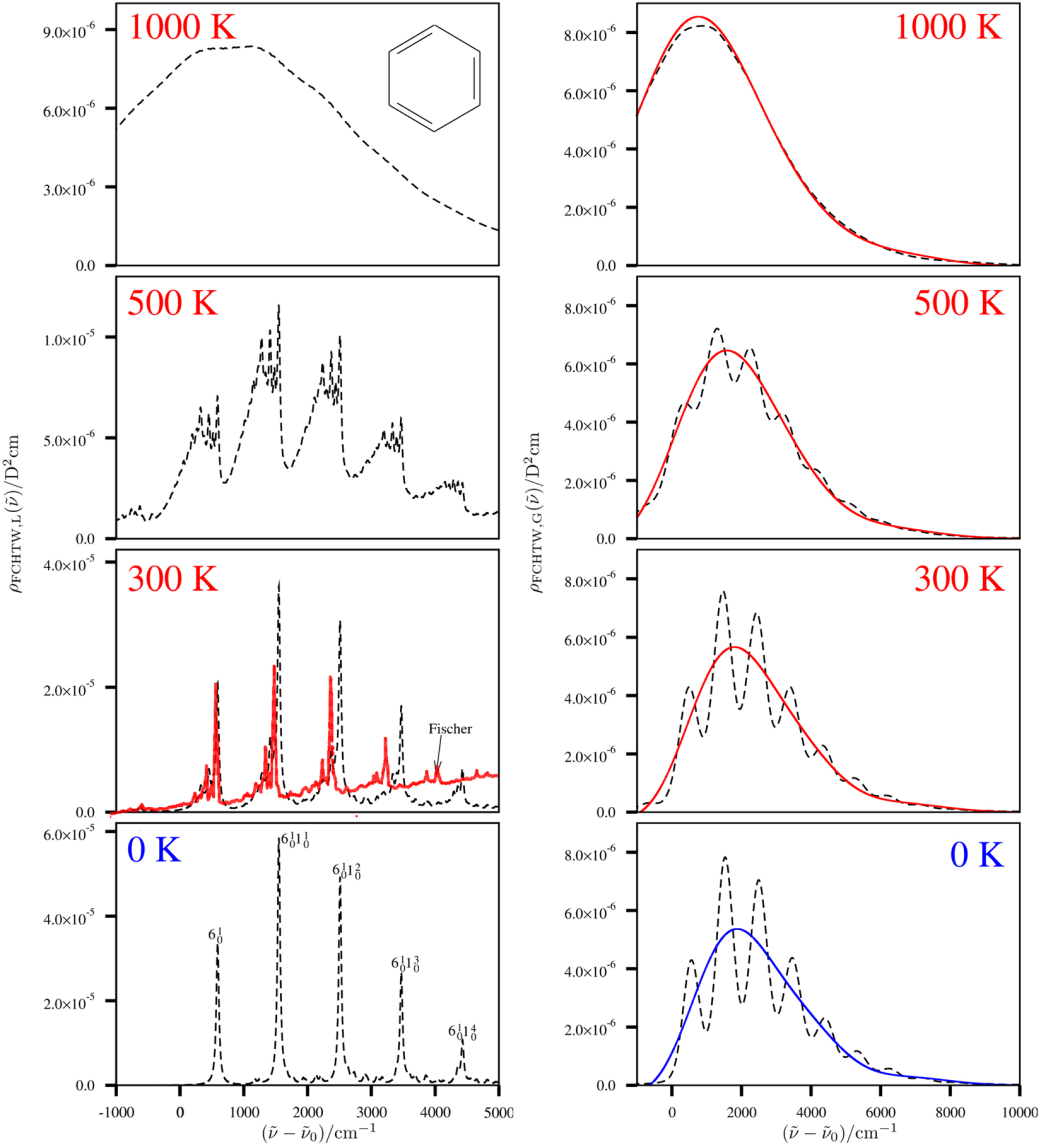
Left part of the figure: The dashed lines are drawn for the TCF-FFT approach with a Lorentzian line shape function with FWHM of 50 cm^−1^. A time increment Δ*t* of 0.51 fs and a grid with 2^16^ grid points are used for the corresponding FFT calculations. The experimental UV absorption spectrum as reported by Fischer in ref.^37^ is additionally shown in red, which has been adapted from ref.^37^ and shifted to match approximately the position of the major peak in the region below the 0–0 transition wavenumber and rescaled to have similar peak height as the one computed for the ${6}_{0}^{1}$ transition. Right part of the figure: The dashed lines are drawn for the TCF-FFT approach with a Gaussian line shape function of with FWHM of 500 cm^−1^. A time increment of 0.10 fs and a grid with 2^15^ grid points are used for the corresponding FFT calculations. Solid lines are drawn for the curve obtained by Edgeworth expansion using up to 4-th order cumulants and, for 1000 K by Edgeworth expansion using up to 8-th order cumulants.

The computed vibronic spectra are shown in Fig. 1. The left hand side in Fig. 1 shows vibronic profiles from TCF-FFT which are convoluted by a Lorentzian line shape function with full width at half maximum (FWHM) of 50 cm^−1^ at temperatures elevating from 0 K to 1000 K. This FC-forbidden vibronic transition is mediated by the non-totally symmetric vibrational modes in the irreducible representation e_2g_ of the D_6h_ molecular symmetry group. The main feature of the vibronic spectrum is from progressions in the totally symmetric C-C stretching mode (963 cm^−1^) building on the so-called false origin from a single excitation of a non-totally symmetric (e_2g_) in-plane bending mode (575 cm^−1^) as indicated in the spectrum at zero Kelvin. The calculated spectrum at 300 K is compared with the experimental data of Fischer^37^. The two spectra agree fairly well in the low energy region but the computed peaks at higher energies are slightly shifted to larger wavenumbers due to the harmonic approximation. As temperature increases the vibrational structure becomes very congested and washed out. At 1000 K (only employed for testing the method) one can not see a resolved vibrational structure any longer, only the corresponding envelope.

On the right hand side of Fig. 1 the two methods (TCF-FFT and CE-Edgeworth) are compared for increasing temperatures. The spectra are convoluted in the TCF-FFT curves (dashed lines) with a Gaussian line shape function of 500 cm^−1^ for FWHM. The second moment (4.51 × 10^4^ cm^−2^ (*hc*_0_)^2^) of the Gaussian line shape function is added to the second moments of the vibronic spectrum to take the line shape function into account (see ref.^29^ for the rationalisation and details). The relatively broad line shape function is used for the TCF-FFT curves to have vibrationally relatively structureless spectra for comparison. At 0, 300 and 500 K, the TCF-FFT curves still show vibrational structure and the CE-Edgeworth curves (solid lines) look like nonlinear regression curves of the corresponding TCF-FFT spectra. When the vibrational structures are also essentially smoothed out in the TCF-FFT curves at 1000 K, the two approaches agree with each other extremely well. Up to the 4-th order cumulants are used for 0, 300, 500 K and up to 8-th order cumulants are computed for 1000 K.

In Table 1 the moments computed numerically (via numerical derivatives) and analytically are compared. At low orders and all temperatures the two methods agree well and for higher orders still the agreement is satisfactory. One of the advantages of the numerical method is that one only needs to compute the TCF for the first few time steps and it can be improved by controlling the time increment and the number of data points. The analytical method usually meets a combinatorial problem in high order cumulant calculations due to the analytic derivatives of the inverse matrix^29^. The second advantage of the numerical method is that it is easy to include linear and nonlinear non-Condon effects. The third advantage is that one can incorporate general line shape functions which would not have well defined cumulants (see the discussion on page 415 of ref.^29^). Lastly, the computational cost of the numerical cumulant expansion method is that the number of data points to be evaluated is almost negligible comparing to the TCF-FFT approach, which is about three orders of magnitude more expensive.

**Table 1:** Analytically and numerically computed moments.

n	${\boldsymbol{\langle }}{{\boldsymbol{E}}}_{\underline{{\boldsymbol{\varepsilon }}}^{\prime} ,\underline{{\boldsymbol{\varepsilon }}}}^{{\boldsymbol{n}}}{\boldsymbol{\rangle }}{\boldsymbol{/}}{\boldsymbol{(}}{\bf{c}}{{\bf{m}}}^{-{\bf{1}}}\,{\boldsymbol{h}}{{\boldsymbol{c}}}_{{\bf{0}}}{{\boldsymbol{)}}}^{{\boldsymbol{n}}}$							
	*T* = 0 K		*T* = 300 K		*T* = 500 K		*T* = 1000 K	
	Analytical	Numerical	Analytical	Numerical	Analytical	Numerical	Analytical	Numerical
1	2.61 × 10^3^	2.61 × 10^3^	2.47 × 10^3^	2.47 × 10^3^	2.12 × 10^3^	2.12 × 10^3^	1.12 × 10^3^	1.12 × 10^3^
2	9.24 × 10^6^	9.21 × 10^6^	8.64 × 10^6^	8.62 × 10^6^	7.38 × 10^6^	7.36 × 10^6^	5.64 × 10^6^	5.63 × 10^6^
3	4.07 × 10^10^	4.05 × 10^10^	3.77 × 10^10^	3.75 × 10^10^	3.17 × 10^10^	3.15 × 10^10^	2.03 × 10^10^	2.01 × 10^10^
4	2.14 × 10^14^	2.12 × 10^14^	1.97 × 10^14^	1.95 × 10^14^	1.66 × 10^14^	1.64 × 10^14^	1.29 × 10^14^	1.27 × 10^14^
5	—	—	—	—	—	—	8.00 × 10^17^	7.82 × 10^17^
6	—	—	—	—	—	—	6.55 × 10^21^	6.33 × 10^21^
7	—	—	—	—	—	—	5.84 × 10^25^	5.54 × 10^25^
8	—	—	—	—	—	—	6.16 × 10^29^	5.71 × 10^29^

4.51 × 10^4^ cm^−2^ (*hc*_0_)^2^ is added to the second moments to take the Gaussian line shape function (FWHM = 500 cm^−1^) into account; see ref.^29^ for details. A time increment of 0.10 fs is used for computing the numerical derivatives.

Mean excitation wavenumbers (${\varepsilon }_{i}^{\prime} \langle {\hat{v}}_{i}^{\prime} \rangle /(h{c}_{0})$ with ${\hat{v}}_{i}^{\prime}$ being a number operator of *i*-th mode in the final electronic state) of individual modes are computed analytically for the HT active e_2g_ symmetric vibrational modes of the final state, and are given in Table 2. The corresponding first derivative in Eq. (6) can be performed numerically or analytically by assigning *z* = diag(1, …, 1) and ${\bf{z}}{\boldsymbol{^{\prime} }}={\rm{diag}}\mathrm{(1,}\,\ldots ,\,\mathrm{1,}\,{{\rm{e}}}^{{\rm{i}}{\varepsilon }_{i}^{\prime} t\mathrm{/(2}\hslash )}\mathrm{,\; 1,}\ldots \mathrm{1)}$ to Eq. 17. The mean excitation energy can serve as a parameter for the individual vibrational degrees of freedom as an effective reorganisation energy or a Huang–Rhys factor (when normalised by its harmonic energy), which can be characterised as a function of structural deformation, frequency change, Duschinsky mode coupling and temperature both in the Condon and non-Condon approximation. One might naively expect a larger mean excitation energy as the temperature increases but the mean values of high frequency modes (1665 and 3389 cm^−1^) in some intermediate temperature ranges are smaller than at zero Kelvin. This can be rationalised as follows: Because the Duschinsky mode mixing between low and high frequency modes is small in the present case, the high frequency modes can not obtain thermal energy from the low frequency modes efficiently. Thus the high frequency modes are almost thermally inactive, whereas the total integrated profile (*ρ*_tot_) increases as temperature increases. In the mean energy calculation of the high frequency modes at finite temperatures the denominator (total intensity) increases because low frequency modes accept thermal energy while the numerator (excitation of high frequency modes) stays constant. Therefore the mean excitation energies of high frequency modes are reduced at finite temperatures. If Duschinsky rotation couples the low and high frequency modes significantly, however, thermal energy can be transfered to the high frequency modes via the low frequency modes in the initial state, accordingly the mean excitation energies of high frequency modes can increase as temperature increases.

**Table 2:** Mean excitation wavenumbers of the components of individual vibrational e_2g_ symmetric modes of benzene as computed for different temperatures.

$${{\boldsymbol{\varepsilon }}}_{{\boldsymbol{i}}{\boldsymbol{^{\prime} }}}{\boldsymbol{\langle }}{\hat{{\boldsymbol{v}}}}_{{\boldsymbol{i}}{\boldsymbol{^{\prime} }}}{\boldsymbol{\rangle }}{\boldsymbol{/}}{\boldsymbol{(}}{\boldsymbol{h}}{{\boldsymbol{c}}}_{{\bf{0}}}\,{\bf{c}}{{\bf{m}}}^{-{\bf{1}}}{\boldsymbol{)}}$$					
Mode *ν*_*i*_	${\tilde{{\boldsymbol{\omega }}}}_{{\bf{e}}}^{\prime}$/cm^−1^	*T* = 0 K	*T* = 300 K	*T* = 500 K	*T* = 1000 K
*ν*_6_	575	2.62 × 10^2^	2.90 × 10^2^	3.79 × 10^2^	7.25 × 10^2^
*ν*_6_	575	2.62 × 10^2^	2.90 × 10^2^	3.79 × 10^2^	7.25 × 10^2^
*ν*_9_	1237	1.60 × 10^1^	1.78 × 10^1^	4.64 × 10^1^	2.51 × 10^2^
*ν*_9_	1237	1.60 × 10^1^	1.78 × 10^1^	4.64 × 10^1^	2.51 × 10^2^
*ν*_8_	1665	2.54 × 10^1^	2.38 × 10^1^	3.09 × 10^1^	1.65 × 10^2^
*ν*_8_	1665	2.54 × 10^1^	2.38 × 10^1^	3.09 × 10^1^	1.65 × 10^2^
*ν*_7_	3389	8.14 × 10^1^	7.49 × 10^1^	6.08 × 10^1^	6.37 × 10^1^
*ν*_7_	3389	8.14 × 10^1^	7.49 × 10^1^	6.08 × 10^1^	6.37 × 10^1^

The numbering used for the modes *ν*_6_, *ν*_7_, *ν*_8_ and *ν*_9_, correspond to that used by Wilson for benzene and translates to *ν*_18_, *ν*_15_, *ν*_16_ and *ν*_17_ in Herzberg’s nomenclature, respectively. The corresponding harmonic vibrational wavenumbers ${\tilde{\omega }}_{{\rm{e}}}^{\prime}$ as computed in ref.^30^ for the electronically excited state and as used in the present calculations are also given.

In closing the section, the moments or the cumulants of the vibronic excitation energy can provide intuitively useful information concerning the vibronic transition profile with almost no computation cost comparing to the TCF-FFT method. Furthermore, the mean excitation energy of individual mode opens a new interpretation for the vibronic transition with a single quantity incorporating the mode mixing and the non-Condon effects as well as the temperature, geometrical change and distortion effects.

## Conclusion and outlook

We have discussed a cumulant expansion method for describing non-Condon transitions and applied it to the prototypical one-photon electric dipole 1 ^1^A_g_ → 1 ^1^B_2u_ transition of benzene, which is FC forbidden but HT allowed in the linear HT approximation. The method is particularly powerful when one does not require all the details of the vibronic structures, but rather only quantities such as peak maximum, mean and variance of the spectral shape. This method is computationally much cheaper than the sum-over-states and time-correlation function approach. Moreover, the information (*e.g*. on the spectroscopically relevant energy window) from the cumulant expansion method can be used in the calculation within the other two methods. For example, the relevant vibronic transition energy domain obtained from the cumulant expansion method can be used for screening the excitation configurations to avoid the unnecessary integral calculations in the time-independent approach. In the time-dependent approach, the same information can be exploited to define via the reciprocal energy window the time increment for the numerical propagation of the wavepacket. The number of discrete time steps with a given time increment determines the desired spectral resolution and the total wavepacket propagation time. Herein we compared a numerical approach for the calculation of cumulants with the results from an analytical scheme. The results obtained numerically are still fairly good. With this method, one can incorporate easily nonlinear non-Condon terms and various line shape functions. In the time-correlation function calculation the real part and imaginary part at each time step provide automatically the even and odd moments, respectively. In the first few time steps we already have the first few moments available and the probability distribution function (information) becomes complete as time progresses. The benzene example selected herein serves to illustrate the principle of the method for future routine applications for molecular systems with hundreds of atoms. As an outlook for the approach described herein, we would like to indicate that it can be generalized for the even more challenging anharmonic vibronic transition problem, because the method has a clear connection to the time correlation function approach. When the time correlation function can be evaluated only for the first few time steps, the cumulants can be determined numerically. The overall computational cost is only the evaluation of a few time correlation function.

## References

[1] Franck J (1925). Elementary processes of photochemical reactions. Trans. Faraday Soc..

[2] Condon EU (1928). Nuclear motions associated with electron transitions in diatomic molecules. Phys. Rev..

[3] Duschinsky FZD (1937). der Elektronenspektren mehratomiger Moleküle. Acta Physicochim. URSS.

[4] Rahimi-Keshari S, Lund AP, Ralph TC (2015). What can quantum optics say about complexity theory?. Phys. Rev. Lett..

[5] Huh J, Guerreschi GG, Peropadre B, McClean JR, Aspuru-Guzik A (2015). Boson sampling for molecular vibronic spectra. Nature Photon..

[6] Huh, J. & Yung, M.-H. Hierarchy in sampling gaussian-correlated bosons. *arXiv:1608.03731* (2016).

[7] Shen, Y. *et al*. Quantum simulation of molecular spectroscopy in trapped-ion device. *arXiv:1702.04859* (2017).

[8] Herzberg G, Teller E (1933). Schwingungsstruktur der Elektronenübergänge bei mehratomigen Molekülen. Z. Phys. Chem. B.

[9] Jankowiak H-C, Stuber JL, Berger R (2007). Vibronic transitions in large molecular systems: Rigorous prescreening conditions for Franck-Condon factors. J. Chem. Phys..

[10] Santoro F, Lami A, Improta R, Bloino J, Barone V (2008). Effective method for the computation of optical spectra of large molecules at finite temperature including the Duschinsky and Herzberg-Teller effect: The *Q*_*x*_ band of porphyrin as a case study. J. Chem. Phys..

[11] Tannor DJ, Heller EJ (1982). Polyatomic Raman scattering for general harmonic potentials. J. Chem. Phys..

[12] Yan YJ, Mukamel S (1986). Eigenstate-free, Green function, calculation of molecular absorption and fluorescence line shapes. J. Chem. Phys..

[13] Ianconescu R, Pollak E (2004). Photoinduced cooling of polyatomic molecules in an electronically excited state in the presence of Dushinskii rotations. J. Phys. Chem. A.

[14] Borrelli R, Capobianco A, Peluso A (2012). Generating function approach to the calculation of spectral band shapes of free-base chlorin including Duschinsky and Herzberg-Teller effects. J. Phys. Chem. A.

[15] Baiardi A, Bloino J, Barone V (2013). General Time Dependent Approach to Vibronic Spectroscopy Including Franck–Condon, Herzberg–Teller, and Duschinsky Effects. J. Chem. Theory Comput..

[16] Huh J, Berger R (2012). J. Phys. Conf. Ser..

[17] Doktorov EV, Malkin IA, Man’ko VI (1979). The dushinsky effect and sum rules for vibronic transitions in polyatomic molecules. J. Mol. Spectrosc..

[18] Cederbaum LS, Domcke W (1977). Theoretical aspects of ionization potentials and photoelectron spectroscopy:a Green’s function approach. Adv. Chem. Phys..

[19] Heller EJ (1978). Quantum corrections to classical photodissociation models. J. Chem. Phys..

[20] Islampour R (1989). Electronic spectral line shape of a polyatomic molecule. Chem. Phys..

[21] Mukamel, S. *Principles of Nonlinear Optical Spectroscopy* (Oxford University Press, New York 1995).

[22] Wadi H, Pollak E (1999). Theory of laser cooling of polyatomic molecules in an electronically excited state. J. Chem. Phys..

[23] Schatz, G. C. & Ratner, M. A. *Quantum mechanics in chemistry* (Dover Publications, Inc., New York 2002).

[24] Liang KK (2003). Influence of distortion and Duschinsky effects on Marcus-type theories of electron transfer rate. Phys. Chem. Chem. Phys..

[25] Lax M (1952). The Franck–Condon Principle and Its Application to Crystals. J. Chem. Phys..

[26] Kubo R, Toyozawa Y (1955). Application of the Method of Generating Function to Radiative and Non-Radiative Transitions of a Trapped Electron in a Crystal. Prog. Theore. Phys..

[27] Tatchen J, Pollak E (2008). Ab initio spectroscopy and photoinduced cooling of the trans-stilbene molecule. J. Chem. Phys..

[28] Huh, J. *Unified description of vibronic transitions with coherent states*. Ph.D. thesis, Johann-Wolfgang-Goethe University, Frankfurt am Main (2011).

[29] Huh J, Berger R (2011). Application of time-independent cumulant expansion to calculation of Franck-Condon profiles for large molecular systems. Faraday Discuss..

[30] Berger R, Fischer C, Klessinger M (1998). Calculation of the vibronic fine structure in electronic spectra at higher temperatures. 1. benzene and pyrazine. J. Phys. Chem. A.

[31] He Y, Pollak E (2001). Theory of cooling of room temperature benzene upon photo-excitation to the S_1_ state. J. Phys. Chem. A.

[32] Coriani S (2010). An atomic-orbital based Lagrangian approach for calculating geometric gradients of linear response properties. J. Chem. Theory Comp..

[33] Berberan-Santos MN (2007). Expressing a probability density function in terms of another PDF: A generalized Gram-Charlier expansion. J. Math. Chem..

[34] Doktorov EV, Malkin IA, Man’ko VI (1977). Dynamical symmetry of vibronic transitions in polyatomic molecules and the Franck-Condon principle. J. Mol. Spectrosc..

[35] Blinnikov S, Moessner R (1998). Expansions for nearly Gaussian distributions. Astron. Astrophys. Suppl. Ser..

[36] Frigo, M. & Johnson, S. G. The design and implementation of FFTW3. *Proceedings of the IEEE* **93**, 216–231 (2005). Special issue on “ProgramGeneration, Optimization, and Platform Adaptation”.

[37] Fischer, G. *Vibronic Coupling* (Academic Press Inc., London 1984).

